# Vitamin A and Pregnancy: A Narrative Review

**DOI:** 10.3390/nu11030681

**Published:** 2019-03-22

**Authors:** Sabina Bastos Maia, Alex Sandro Rolland Souza, Maria de Fátima Costa Caminha, Suzana Lins da Silva, Rachel de Sá Barreto Luna Callou Cruz, Camila Carvalho dos Santos, Malaquias Batista Filho

**Affiliations:** 1 Maternal and Child Healthcare Postgraduate Program, Instituto de Medicina Integral Prof. Fernando Figueira (IMIP), Recife 50070-550, Pernambuco, Brazil; alexrolland@uol.com.br (A.S.R.S.); fatimacaminha@imip.org.br (M.d.F.C.C.); suzanalinsilva@gmail.com (S.L.d.S.); malaquias.imip@gmail.com (M.B.F.); 2 Department of Obstetrics and Gynecology, Lauro Wanderley University Hospital, Federal University of Paraíba (UFPB), João Pessoa 58059-900, Paraíba, Brazil; 3 Department of Maternal and Child Healthcare, Federal University of Pernambuco (UFPE), Recife 50670-901, Pernambuco, Brazil; 4 Biological and Health Sciences Center, Catholic University of Pernambuco (UNICAP), Recife 50050-900, Pernambuco, Brazil; 5 Coordination of the Nursing Mentoring Program, Faculdade Pernambucana de Saúde (FPS), Recife 51180-001, Pernambuco, Brazil; 6 Department of Nursing, Regional University of Cariri (URCA), Crato 63105-000, Ceará, Brazil; rachel.callou@hotmail.com; 7 Research Directorate, IMIP, Recife 50070-550, Pernambuco, Brazil; camilacarvalhoupe@gmail.com

**Keywords:** Vitamin A, vitamin A deficiency, pregnancy

## Abstract

Vitamin A is a crucial micronutrient for pregnant women and their fetuses. In addition to being essential for morphological and functional development and for ocular integrity, vitamin A exerts systemic effects on several fetal organs and on the fetal skeleton. Vitamin A requirements during pregnancy are therefore greater. Vitamin A deficiency (VAD) remains the leading cause of preventable blindness in the world. VAD in pregnant women is a public health issue in most developing countries. In contrast, in some developed countries, excessive vitamin A intake during pregnancy can be a concern since, when in excess, this micronutrient may exert teratogenic effects in the first 60 days following conception. Routine prenatal vitamin A supplementation for the prevention of maternal and infant morbidity and mortality is not recommended; however, in regions where VAD is a public health issue, vitamin A supplementation is recommended to prevent night blindness. Given the importance of this topic and the lack of a complete, up-to-date review on vitamin A and pregnancy, an extensive review of the literature was conducted to identify conflicting or incomplete data on the topic as well as any gaps in existing data.

## 1. Introduction

Vitamin A was discovered 106 years ago [1] and has been recognized as a public health priority by the World Health Organization (WHO) for more than six decades [2]. Nevertheless, many aspects of vitamin A deficiency (VAD), such as its epidemiology, classification, and even its metabolism and pathophysiology, are still not fully understood. The aim of this review article, which focuses on the pregnant woman and her fetus as representing the most vulnerable group insofar as this problem is concerned [3,4,5], is to contribute towards clarifying these issues and identifying possible new alternatives regarding practical actions, including future research requirements.

Vitamin A plays an important role in ocular function, as it is involved in cell differentiation, in the maintenance of eye integrity, and in the prevention of xerophthalmia. Its deficiency is the main cause of preventable blindness worldwide [4]. Vitamin A is also associated with bone development, has a protective effect on the skin and mucosa, plays a vital role in the functional capacity of reproductive organs, participates in strengthening the immune system, is related to the development and maintenance of epithelial tissue, and contributes to the development of normal teeth and hair [6,7,8]. In addition to its important role in various body tissues [6], vitamin A is essential to the normal development of the embryo [9].

Pregnancy is a period of specific nutritional needs for maintaining the health of both the mother and the fetus. During this period, there is an increase in the demand for vitamin A, particularly in the third quarter because of the accelerated fetal development in this phase [10]. According to the WHO, VAD is still considered a public health issue at a population level, particularly in some developing countries, affecting approximately 19 million pregnant women [4].

In contrast, due to the possible teratogenic effects associated with high doses of vitamin A [11], excessive intake of this vitamin is a concern, principally in developed countries. The main adverse effects associated with excessive vitamin A intake, particularly at the beginning of the first quarter of pregnancy, are congenital malformations involving the central nervous and cardiovascular systems and spontaneous abortion [12,13].

Therefore, adequate vitamin A levels during pregnancy are essential for the health of both the mother and the fetus. No general review of recent evidence on vitamin A and pregnancy has been published. Therefore, the present study proposes to summarize available knowledge on vitamin A metabolism, epidemiological data on the nutritional status of vitamin A in pregnant women, and the importance of supplementation, including current recommendations.

This narrative literature review was conducted between March and December 2018 and updated in January 2019. A search was made of the PubMed, ScIELO, and LILACS databases using the following Medical Subject Headings (MeSH): “Vitamin A” or “Vitamin A Deficiency” and “Pregnancy”. The search was limited to the preceding ten years and to human studies. The titles and abstracts of the identified articles were read, and those concerning vitamin A/retinol and pregnancy were included. Only articles written in English, Portuguese, or Spanish were included. Articles involving topics clearly not relevant were excluded. The selected articles were read in full and further articles identified from their references were also reviewed with a view to including classic and critical studies that may have been missed in the initial search. A manual search was also made for reports from major micronutrient conferences. A total of 144 references were thus used in the present review.

## 2. Vitamin A: Summary Review of Metabolism

The metabolism of vitamin A is complex and involves different forms, sources, and mechanisms such as carrier proteins, enzymes, storage, and physiological and pathological complications [6]. In humans, vitamin A has three active forms (retinal, retinol and retinoic acid) and a form of storage in the liver (retinyl ester) [14]. This liposoluble micronutrient is not synthesized by the body and must be obtained through the diet. It is available from two main sources: preformed vitamin A (retinol and retinyl ester) and provitamin A (caratenoids) [15,16,17]. Of the numerous naturally occurring carotenoids, beta-carotene, alpha carotene, and beta-cryptoxanthin are major provitamin A carotenoids present in foods [18].

Preformed vitamin A is found in foods of animal origin such as dairy products (e.g., milk, yoghurt, and cheese), liver, fish oils, and human milk. Provitamin A, from vegetable sources, is found in fruits, leaves, and tubers such as carrots, pumpkin, kale, spinach, sweet potato, papaya, mango, and red palm oil [14,17,19,20]. Brazilian buriti (*Mauritia vinifera*) and palm oil (*Elaeis guineensis*) represent the richest sources of provitamin A in Brazil [21]. The absorption of vitamin A from vegetable sources is considered poor, and foods of animal origin may be necessary to achieve adequate levels in the body [15,16].

The digestion and absorption of vitamin A are associated with the absorption of lipids. Therefore, critically low dietary fat content (less than 5–10 g/day) or conditions such as pancreatic and hepatic diseases and frequent gastroenteritis that interfere with the digestion or absorption of lipids, resulting in steatorrhea, can interfere with the absorption of vitamin A [14,22,23].

With regard to provitamin A, mammals use both carotenoid oxygenases, β-carotene-15,15′-oxygenase (BCO1), and β-carotene-9′,10′-oxygenase (BCO2) to synthesize retinoids from provitamin A carotenoids. Cleavage by BCO2 produces apocaretenoids, which are converted into retinoids by BCO1. Furthermore, β-cryptoxanthin plays an important role in vitamin A production by limiting competition between the metabolites of β-carotene based on the substrate specificity of BCO2 for carotenoids with 3-OH-α-ionone ring sites. This is also evident from the occurrence of β-cryptoxanthin accumulation in BCO2-deficient mice [24]. The retinol absorbed can be released directly into the extrahepatic tissues or captured by the liver, where it can be stored or released back into the bloodstream to meet the body’s requirement [25]. The liver reserve may be able to fulfill the required demands for a long period of time (up to months) [14].

In any tissue, including the liver, vitamin A is converted to retinoic acid, which is the active metabolite required for proper morphogenesis. Unlike retinol, retinoic acid is not a stable metabolite, being present in very low levels in serum [26]. High concentrations of certain metabolites of retinoic acid (trans-retinoic acid and 13-cis-retinoic acid) can influence gene activity during critical periods of organogenesis and embryogenesis, leading to teratogenicity [27,28].

Circulating vitamin A is transported in the plasma in a 1:1 complex with retinol-binding protein (RBP). The retina and other tissues dependent on vitamin A have specific binding sites for RBP and vitamin A. As RBP is the only carrier [29], conditions that reduce its levels such as proteinuria, kwashiorkor (protein malnutrition), and zinc deficiency may contribute to VAD. For example, pre-eclampsia, which evolves with proteinuria [30], may cause a decrease in serum retinol levels [31,32].

The transfer of vitamin A from mother to child occurs via the placenta during gestation and at birth and via the mammary gland during lactation (breastfeeding). During pregnancy, due to the decrease in serum retinol levels in pregnant women (particularly in the third quarter) and the selective placental barrier, the newborn hepatic reserves of vitamin A are low at birth to avoid possible teratogenic effects [33]. After birth, a major part of serum retinol is transported to the breast by RBP, reaching the breast milk [34]. From then on, the transport of vitamin A to breast milk in the first six months of life provides 60-fold more vitamin A when compared with the placental route during the entire pregnancy [35]. Furthermore, breast milk also transports active provitamin A carotenoids, which serve as additional nutrients for the infant. Despite the importance of carotenoids in promoting the health of breast-feeding mothers and their newborns, in recent studies conducted with rats in early life (during breast-feeding), excess vitamin A intake has been associated with obesity [36,37].

## 3. Vitamin A Deficiency during Pregnancy: Epidemiological Aspects

Despite extensive understanding of the pathophysiology of VAD, with its signs and symptoms being well recognized by health professionals and a proportion of the general population, VAD remains among the major collective health priorities in the world today, together with iron-deficiency anemia and iodine deficiency [38]. Regardless of national and international agreements involving policy makers, program managers in health and education, and multisector programs of action (e.g., agricultural policies, food supply and enrichment of industrialized foods with specific nutrients, and selective distribution of basic food baskets), epidemiological control of VAD remains a challenge in Brazil and in several other developing countries [4].

Regrettably, basic data for the development, implementation, and evaluation and monitoring of policies and programs (public or private) are therefore unavailable at the consistency required to resolve the problem at the population level. For example, and principally as an object of study, the important issue of VAD in pregnant women is without doubt the most obscure link in the chain of epidemiological events related to this specific nutritional deficiency at the national and international level.

Conceptually, the entire population may be exposed to the problem at any stage of the biological cycle, from embryonic/fetal life until old age. Nevertheless, it is in the short period of pregnancy/lactation that the risk of this deficiency increases [3,4,5]. This process is fundamentally triggered by biological factors: during pregnancy, there is a considerable increase in nutritional vitamin A requirements due to the double demand from the mother and her child; during lactation, exclusively breastfeeding should be the autonomous and complete source of fluids, energy, and nutrients for the infant [14]. Nevertheless, not only should the physiological nutritional demands of children in the first months and years of life be taken into consideration, but also the impact of these demands on the health/illness process that may continue into adult life, including preventing and delaying the onset of chronic noncommunicable diseases [39].

Pregnancy represents a nutritionally crucial period, either because of previous deficiencies being reduced or aggravated by the gestational process, or because excesses and inadequacies of nutrients in the diet may produce specific nutritional disorders [40]. Thus, nutrients including vitamin A can interfere with the occurrence or worsening of previous or coexisting diseases during pregnancy, childbirth, and in the postpartum.

VAD is more frequent in the last quarter of pregnancy because of the physiological increase in maternal blood volume and accelerated fetal development in the final phase of pregnancy [41,42]. Pregnant women may be more prone to developing VAD during periods in which there is a shortage of foods rich in vitamin A or in the presence of infections, diabetes mellitus, or gestational diabetes [43,44,45].

The WHO defines VAD in pregnancy as serum retinol levels of <0.70 µmol/L, with the condition being considered a serious public health issue when the prevalence occurs in 20% or more of pregnant women; moderate when affecting between 10% and 20%; and mild when between 2% and 10% [4,46,47] (Table 1). It is noteworthy that serum retinol levels should ideally be analyzed using high performance liquid chromatography (HPLC) [48].

However, the value of serum retinol as a marker of vitamin A status during pregnancy (especially in the last quarter) and during inflammation has been questioned [49]. The relation between hepatic reserves and circulating retinol is altered due to the physiological changes during pregnancy. Moreover, RBP, the retinol transporter protein in the serum, is an acute phase protein and its concentration may alter during inflammation [49,50]. Physiologically, gestation itself represents an inflammatory process in view of the immunological adaptations necessary to ensure the viability of the conceptus [51,52]. Thus, serum retinol and its conventional cut-off point may be underestimating the status of vitamin A at the end of pregnancy and in populations with a high prevalence of infections [49].

Accordingly, studies that assessed the nutritional status of vitamin A during pregnancy revealed a negative correlation between serum retinol and C-reactive protein (CRP) [49,53]. In a meta-analysis, Thurnham et al. concluded that the increase in CRP is associated with a decrease of 25% in the serum retinol level [54]. CRP is an acute phase protein, which is mainly a part of the innate immune response, and its elevation may occur during infections and in many other situations including a physiological pregnancy [51,52]. In view of the evidence that the infectious process is associated with a transitory reduction in the biomarkers of vitamin A status, the Biomarkers Reflecting Inflammation and Nutritional Determinants of Anemia (BRINDA) project has suggested approaches for correcting for inflammation using both retinol and RBP when estimating VAD in children and women [55,56].

Because night blindness is the main clinical consequence of VAD, the International Vitamin A Consultative Group (IVACG) [57] recommended that a night blindness prevalence of ≥5% during pregnancy, as observed in population studies, is sufficient to classify VAD as a public health issue [57]. In several countries such as Brazil, there are still no conclusive data on the nutritional status of vitamin A in pregnant women. In practice, in areas where night blindness is common, VAD is still often seen as widespread [58].

At the population level, VAD is considered a public health issue in many developing countries, despite the fact that the lack of data does not permit WHO recommendations to be drafted. Between 1995 and 2005, VAD affected approximately 19 million pregnant women, with an estimated 9.8 million pregnant women worldwide affected by night blindness, the majority being from Africa and South-East Asia [4].

Socioeconomic factors play an important role in VAD and in most deficiencies of epidemiological interest since they are more prevalent in poor countries, regions, and underserved families affected by inequalities in income, education, housing, access to health services, and other related aspects [5]. Therefore, the fundamental role played by these factors justifies the WHO criterion of gathering and systematizing information on the situation of vitamin A in countries with gross domestic product (GDP) below 15 thousand dollars [4].

Few studies evaluating the nutritional status of vitamin A in pregnant women have been published over the past ten years, and very few have used a representative sample. Of these, one that can be considered standard and which evaluated 1209 pregnant Chinese women concluded that VAD is a mild public health issue in China. It has been suggested that the low frequency of VAD found in pregnant Chinese women could be explained by improvements in socioeconomic conditions. The report also identified the following risk factors for the occurrence of VAD in pregnant women: residing in poor rural areas; poor educational level; lack of adequate health conditions; low income; advanced gestational age; and unhealthy lifestyle habits such as smoking and alcohol consumption [59].

Another study performed in 319 pregnant and lactating women in the Republic of the Congo showed that VAD is a serious public health issue in that country [60]. Similarly, a review concluded that there was a substantial number of pregnant women with VAD in developing areas of South Asia on account of poverty and socioeconomic limitations [61]. Other evaluations conducted in developing countries also found a high prevalence of VAD in pregnant women: 24.6% of a sample of 3270 pregnant women in Iran [62]; 20% of 80 in Egypt [63]; 18.5% of 200 in Bangladesh [64]; 15.8% of 101 in Nigeria [65]; 13.8% of 738 in Guinea-Bissau [49]; and 10.6% of 160 pregnant adolescents in Venezuela [66]. As can be seen, population samples were statistically robust only in China, Iran, and in Guinea-Bissau.

A recent review that included studies conducted in Ethiopia, Kenya, Nigeria, and South Africa showed a prevalence of VAD that ranged from 21% to 48% among pregnant women [67].

In studies conducted in Brazil to assess the nutritional status of vitamin A during pregnancy and postpartum, the majority of the women were in the postpartum [68,69,70,71,72,73,74,75], which could represent a conceptual bias in terms of sampling. Generally, without focusing specifically on pregnant women, VAD is considered a public health issue in Brazil [76,77,78]. The prevalence of VAD in a group of mothers and their infants in the state of Pernambuco, northeastern Brazil was 6.9% in 664 mothers and 16.1% in 790 children under five years of age [5].

Furthermore, a study conducted in Brazil in the upper Jequitinhonha valley of Minas Gerais reported night blindness in 8.7% of 92 pregnant women interviewed [79]. In another assessment, the prevalence of night blindness was 9.9% among 606 pregnant and postpartum women in Rio de Janeiro [80]. Another study found a prevalence of VAD of 34.8% in 89 pregnant adolescents in the state of Piauí, northeastern Brazil, suggesting the existence of a severe public health issue [53]. In that case, the risk factors were inadequate sanitary conditions and low pre-gestational body mass index (BMI). It was also found that the more advanced the trimester of pregnancy, the lower the serum retinol level, favoring the occurrence of VAD [53].

In one recent study, the prevalence of VAD was found to be 6.2% in 676 pregnant women receiving prenatal care at a reference maternity hospital in northeastern Brazil, hence evaluated as constituting a mild public health issue. In that study, vitamin A deficiency was associated with the third trimester of pregnancy and with maternal anemia. Serum retinol had no statistically significant effect on the infectious process evaluated by CRP; therefore, it was impossible to adjust for inflammation. In view of these findings, further studies should be conducted to evaluate inflammatory markers in accordance with the recommendations of the WHO and of BRINDA [81].

In Brazil, the available data are still insufficient to establish the prevalence and severity of VAD during pregnancy at national, regional, and microregional levels; therefore, further studies are required.

## 4. Vitamin A and Pregnancy: Importance and Effects of Deficiency and Excess

The more common scenario involving VAD occurs when there is an acutely reduced dietary intake of vitamin A, or when there is a prolonged period of dietary scarcity, or a simultaneous combination of these two conditions, i.e., both prolonged and severe, with the possible mediation of an underlying disease [4,82]. VAD can lead to subclinical disorders such as impaired iron mobilization, altered cellular differentiation, and decreased immune response, or clinical disorders such as increased infectious morbidity, growth retardation, anemia, and xerophthalmia [83].

The term xerophthalmia is used to designate the ocular manifestations of VAD. These ocular manifestations also include night blindness caused by corneal ulceration and keratomalacia [83]. Night blindness is one of the first manifestations of this specific micronutrient deficiency, although not a pathognomonic signal as it can also occur in retinitis pigmentosa [84,85].

Vitamin A is important for the pregnant woman and for the fetus, being essential for the maintenance of maternal night vision and fetal ocular health besides the development of other organs and the fetal skeleton and maintenance of the fetal immune system [17,63,86,87]. Maternal and infant concentrations of vitamin A compounds have been associated with neonatal outcome [88]. In this section, the effects of maternal vitamin A levels during pregnancy on fetal and perinatal health are discussed, with a focus on studies published in the last decade together with the classical references on the theme.

There is strong evidence from animal studies that VAD is associated with adverse effects on offspring during the embryonic and post-natal period [1,89,90,91]. From the moment of the formation of the primitive heart and circulatory system and specification of the rhombencephalon, there is already a need for vitamin A. During this critical time, VAD results in severe abnormalities, including early embryonic death. The need for vitamin A in more advanced stages of development is also evident in experimental rodent models. The main target tissues of VAD include the heart, the central nervous system and its derived structures, the circulatory, urogenital and respiratory systems, and the skull, skeleton, and limbs [90]. Recently, a study in rats showed that dietary vitamin A deficiency two weeks before and during pregnancy can result in anorectal malformations and that the development of the enteric nervous system may be affected by the pathological changes involved in these malformations [92].

Studies in humans suggest that low or excessive levels of vitamin A in the diet during pregnancy can result in adverse effects on the fetus [9]. Thus, a recent study evaluated 1180 pregnant women in the first trimester and observed that 48 newborns presented congenital malformations. The serum concentrations of selenium, zinc, magnesium, and vitamins A, E, B12, and folic acid were assessed, and were significantly lower in mothers of newborns with congenital malformations than in the mothers of newborns without malformations, thus highlighting a possible association between congenital malformations and VAD [93]. These observations led a group of investigators at the Instituto de Medicina Integral Prof. Fernando Figueira (IMIP), in the state of Pernambuco, Brazil, to follow a cohort of pregnant women over a year ago, in view of the outbreak of microcephaly that reached its peak in January/February 2016.

Maternal VAD is possibly one of the main causes of fetal growth restriction and of the subsequent risk of insulin resistance and glucose intolerance in adulthood [94,95]. Studies suggest that VAD is associated with diabetes mellitus and gestational diabetes [45]. VAD during pregnancy can affect the development of the endocrine pancreas in rats [95], suggesting a possible role of VAD in the pathogenesis of diabetes [96]. A recent study in mice explained why VAD during pregnancy can hamper the development of the endocrine pancreas [96].

Studies in animals showed abnormal fetal inner ear development resulting from maternal vitamin A deficiency during pregnancy [97,98,99]. Thus, it is believed that adequate levels of vitamin A during pregnancy can promote the normal development of the inner ear and reduce the risk of sensorineural hearing loss in humans [100]. These hypotheses are also based on the evidence of an increased risk of otitis media associated with VAD [101], with studies suggesting that pre-school supplementation with vitamin A can reduce the risk of hearing loss caused by otitis media [102].

VAD in the second trimester of pregnancy was found to be associated with a three-fold increased risk of schizophrenia and other schizophrenia spectrum disorders in children in a large cohort study including 19,044 live births [103].

In another recent cohort study, the size of offspring bones and growth at birth were evaluated in mother-child pairs, with the retinol serum levels of 520 mothers being negatively associated with these measures, while the serum beta-carotene levels of 446 mothers were positively associated with these measures [104].

According to a case-control study including 31 pregnancies diagnosed with congenital diaphragmatic hernia (CDH) and 46 control pregnancies, there is also evidence that dietary intake of vitamin A during pregnancy below the recommended daily intake is significantly associated with an increased risk of fetuses having CDH. Notwithstanding, the assessment of maternal vitamin A intake was through a food frequency questionnaire [105].

In a study with animals, severe VAD during pregnancy was associated with fetal renal agenesis [89], whereas mild VAD led to a decrease in kidney weight and the number of nephrons in the newborn [106]. Based on these findings, a study was conducted in 16 mothers with VAD and 64 mothers with vitamin A sufficiency. Newborn babies of the mothers with VAD had significantly lower mean retinol levels and the dimensions of both kidneys were smaller than those of the newborns of mothers with vitamin A sufficiency [63]. A recent systematic review found an association between vitamin A deficiency in pregnancy and a negative effect on kidney function and kidney structure in the child [107].

As stated above, maternal VAD is associated with several negative effects in the offspring. In contrast, excess vitamin A has teratogenic effects as shown in several species of animals [108], with the type of malformation depending on the level of vitamin A and the gestational stage at which vitamin A is administered [109]. Given the teratogenicity of vitamin A in animals and of isotretinoin in humans, vitamin A (not beta-carotene) has been considered teratogenic, particularly during the first 60 days following conception in humans [12,110]. Isotretinoin is a drug that contains one of several derivatives of vitamin A, 13-cis-retinoic acid, which is much used to treat dermatological conditions, particularly cystic acne and nodular acne. It is considered teratogenic and is contraindicated during pregnancy [111,112].

The mechanism of action by which vitamin A exerts teratogenicity is attributed to the influence of high concentrations of certain retinoic acid metabolites (such as trans-retinoic acid and 13-cis-retinoic acid) on the function of genes during critical periods of organogenesis and embryogenesis [27,28].

Concern regarding the teratogenicity of vitamin A in humans began with the study conducted by Rothman et al. [110], which concluded that a total vitamin A intake in pregnant women of over 15,000 IU (4500 μg retinol equivalents [RE]) per day in the diet or more than 10,000 IU (3000 μg RE) in the form of supplements increases the risk of abnormalities in the development of neural crest tissues (on which 13-cis-retinoic acid has a teratogenic effect). Intakes of this order are not rare in the populations of high-income countries whose habitual diet contains higher levels of vitamin A than those recommended and who often consume vitamin supplements and/or foods rich in preformed vitamin A such as liver [113].

There is little published information on the doses of vitamin A that pose a risk to women of childbearing age or at different stages of pregnancy. When the dose of preformed vitamin A is above 10,000 IU per day, there may be a potential risk of teratogenicity. There are reports of malformations in children when their mothers consume high doses of preformed vitamin A during pregnancy (>25,000 IU/day). These reports highlight anomalies of the urinary tract [28,113].

The increase in preformed vitamin A (retinoic acid) in maternal blood during the first quarter of pregnancy is associated with miscarriage and congenital malformations involving the central nervous and cardiac systems [12,13]. Thus, given the risk of cardiac malformation, an intake of retinol exceeding 10,000 IU per day during pregnancy is considered a risk factor for fetal cardiopathy (absolute risk between 1% and 2%), suggesting an indication for fetal echocardiography during the prenatal period [114].

## 5. Vitamin A Supplementation during Pregnancy: Recent Evidence and Current Recommendations

### 5.1. Recent Evidence

The three strategies for the prevention and control of VAD according to the WHO are as follows: (1) supplementation with massive doses as an emergency measure in the short term; (2) fortification of foods (redistribution of nutrients) in the medium term; and (3) dietary diversification as a definitive solution in the long term [46].

Programs for the distribution of massive doses usually attain good results; however, over time, they become ineffective because they depend on the active participation of the community and political interest. Dietary diversification, as an ideal solution, is difficult and slow since it involves changing habits and the consumption of specific foods. Accordingly, the strategy that remains is the fortification of foods, which consists of increasing the nutrient content in certain foods, involving several essential elements such as a food vector, nutrient aggregation in a manner that ensures its stability until reaching the consumer, the integrity of the organoleptic characteristics of the vector, and a simple and low-cost fortification technology [46].

During pregnancy, the most recent evidence is related to vitamin A supplementation. When this strategic alternative was applied to women before, during, and after pregnancy in a population with chronic VAD, children’s pulmonary function improved in a large cohort of 9 to 13-year-old children in rural Nepal whose mothers had participated in a placebo-controlled, double-blind, cluster-randomized trial of vitamin A or beta-carotene supplementation [115]. The benefit of maternal supplementation with vitamin A was limited to children whose mothers received preformed vitamin A and was not seen in those whose mothers received beta-carotene, possibly because beta-carotene is a less efficient source of vitamin A than the preformed ester [115].

A cohort study of rural Bangladeshi children from two previous trials showed that vitamin A supplementation in women during prenatal and postnatal periods is associated with an improvement in school performance and aspects of executive function in children assessed at 8 years of age, while general intelligence, memory, and motor functions are not affected by prenatal or postnatal supplementation with vitamin A [116].

In relation to food fortification, a recent study in Denmark suggested an association between fetal exposure to an increase of 25% in the amount of vitamin A in margarine ingested by the mother during pregnancy and a reduced risk (OR = 0.88) of type 2 diabetes in the offspring during adulthood [117].

Some studies suggest a reduction in the risk of infections after vitamin A supplementation as a consequence of its important function in the immune system, improving host defenses [58,118]. Secretory IgA contributes to intestinal barrier function, with evidence suggesting that these antibodies are involved in immunological homeostasis. Its production depends on IgA antibody-secreting plasma cells and their immediate precursors (plasmablasts), which accumulate in the mucosa [119,120]. All-trans retinoic acid is a metabolite of vitamin A that plays an important role in the immune responses of the intestinal mucosa and it also acts in the feedback loop for the production of enzymes involved in its own synthesis, increasing mucosal IgA responses and enhancing the effectiveness of oral vaccines [119]. However, clinical trials that evaluated vitamin A supplementation in pregnant women showed no reduction in the risk of placental malaria and adverse events during pregnancy [121]. Similarly, another clinical trial that assessed the incidence of malaria among HIV-infected Tanzanian women concluded that vitamin A supplementation did not alter the incidence of malaria during the study [122].

Observational studies conducted in sub-Saharan Africa have suggested that low levels of vitamin A in pregnant women infected with HIV are associated with a significantly increased rate of vertical transmission [123,124] and infant mortality [124,125]. However, a systematic review concluded that vitamin A supplementation during the prenatal or postnatal period probably has little or no effect on the transmission of HIV from mother to child [126].

A recent study reported that vitamin A supplementation during pregnancy increased hemoglobin concentrations and reduced the occurrence of anemia [127]. The improvement of serum retinol levels and the reduction of anemia define an important relationship, as anemia is the most common deficiency of pregnancy [128,129,130]. VAD is, therefore, one of the main causes of anemia; however, the pathogenesis of this relationship remains to be clarified. Vitamin A is known to exert an effect on hematopoiesis, to increase immunity to diseases (thus preventing anemia from infection), and to play a role in the modulation of iron metabolism [131].

In Brazil, a recent retrospective cross-sectional study concluded that the use of multivitamin supplements containing vitamin A during pregnancy prevents VAD regardless of the source administered [132]. The other Brazilian study available on the impact of vitamin A supplementation included only postpartum women [133].

Regarding the diversification of diet as a means of preventing and controlling VAD, a cohort study conducted in Rio de Janeiro to assess the impact of nutritional prenatal care found that the ingestion of a medium-sized piece of ox liver (110 g) per week by pregnant women resulted in a significant reduction in the prevalence of night blindness during follow-up [134].

According to a systematic review conducted in 2015, vitamin A supplementation during prenatal care failed to reduce maternal or perinatal mortality. However, most of the studies analyzed during the review included different populations in relation to the basal level of vitamin A, with no information concerning vitamin deficiency. In addition, there were difficulties in the follow-up of these women. The review suggests that supplementation in HIV-positive pregnant women and those living in vitamin A-deficient areas may reduce nocturnal blindness and anemia, but it is unrelated to the reduction in vertical HIV transmission. A reduction in maternal infection rate has also been suggested; however, it is important to bear in mind that these data are not of good quality [58].

### 5.2. Current Recommendations

According to the 2013 WHO guideline [10], routine supplementation of vitamin A in the prenatal period to prevent maternal or perinatal morbidity and mortality is not recommended. However, in places where VAD is a public health issue, vitamin A supplementation in pregnant women is recommended to prevent night blindness [10].

During pregnancy, there is an increase of approximately 10% to 20% [33] in the need for vitamin A, with the recommended dose being 800 µg/day. It may be difficult to obtain this dose through diet alone, particularly in populations affected by VAD [14].

For prenatal care, vitamin A is available in several formulations. When administered alone, the most commonly used components are retinyl palmitate and retinyl acetate in the form of pills or oil-based solutions. There are other alternatives such as oil, fish liver oil, beta-carotene, and a combination of beta-carotene and vitamin A. The schemes suggested for vitamin A supplementation in pregnant women for the prevention of night blindness in areas with a severe public health problem related to vitamin A are detailed in Table 2 [10]. Food diversification and fortification is also recommended along with supplementation to improve the intake of vitamin A [10].

Because of the teratogenic effects secondary to excessive vitamin A intake, the WHO recommends as safe during pregnancy a maximum dose of up to 10,000 IU daily or 25,000 IU weekly after the first 60 days of gestation [10,12,38].

The symptoms of acute vitamin A toxicity include headache, blurred vision, dizziness, vertigo, nausea, vomiting, and reduced motor coordination secondary to intracranial hypertension. Other symptoms reported are skin peeling, weight loss, and fatigue [10,135]. These toxic effects are usually the result of excessive ingestion of dietary vitamin A supplements. However, regular intake of liver, although generally not a problem in areas with retinol deficiency, can also cause toxicity owing to its high content of vitamin A [10,136].

For developed countries and places where there is no VAD, the UK National Institute for Health and Clinical Excellence (NICE) guidelines for the prenatal period recommend avoiding vitamin supplements containing more than 5000 IU (1500 µg) of vitamin A because of the potential adverse effects, particularly teratogenic effects, associated with high doses [137]. Notwithstanding, most supplements contain beta-carotene instead of retinol, and a high intake of beta-carotene was not found to be associated with congenital defects [11,138]. In addition to these recommendations, the Finnish Food Safety Authority and the National Health Service recommend avoiding the consumption of liver during pregnancy given that it is rich in vitamin A [136,139,140].

Additionally, pregnant women who tend to consume liver are instructed to check the food composition databases of their region, since there can be significant variations in vitamin A levels in liver. For example, in the United States Department of Agriculture’s national nutrient database [141], the vitamin A content of liver and liver products varies from 4900 IU in one raw chicken liver to 59,500 IU in 3 ounces of cooked New Zealand ox liver.

In Brazil, there are no specific guidelines or programs for vitamin A supplementation during pregnancy. A previous decision of the Brazilian Ministry of Health to recommend the administration of massive doses of vitamin A (200,000 IU) for postpartum women being discharged from hospital as a strategy to recompose the tissue reserves of this nutrient depleted during pregnancy and required to meet the increased demands during breastfeeding [142] was implemented in the 1980s but terminated in 2016 in a technical addendum.

Despite the lack of studies conducted to evaluate the vitamin A nutritional status of pregnant women and even in the general population worldwide, vitamin A supplementation has been introduced in several countries [143]. Scientific evidence is required on the prevalence of vitamin A deficiency and on the supplementation and fortification of this micronutrient to enable new guidelines and management strategies to be implemented for pregnant women, for children, for other biologically or socially vulnerable groups, and even for the general population [143,144].

## 6. Conclusions

According to current evidence, adequate levels of vitamin A during pregnancy are of critical importance for the health of pregnant women and their fetuses. Unfortunately, to date, VAD in pregnancy is considered a public health issue. In the past decade, few studies have assessed the nutritional status of vitamin A in pregnant Brazilian women, and further studies with new approaches and new designs should be performed to assess the real magnitude of this problem, particularly in developing countries.

Previous publications reporting a prevalence of 5.2% [59] and 6.2% [81] for serum retinol levels below 0.70 µmol/L may suggest that VAD in pregnant women could finally be close to coming within epidemiological control. Although this is a promising prospect, in view of its importance in decision-making with regard to the readjustment of policies and management of programs on the issue, it should be re-evaluated in new assessments conducted in accordance with WHO and BRINDA recommendations.

During the prenatal period, the current recommendation is that vitamin A supplementation be reserved for the prevention of night blindness in populations with a severe deficiency of this micronutrient. Further research is needed on the dose and duration of vitamin A supplementation during pregnancy. In contrast, in places where VAD is rare, the recommendation is for caution with regard to excess dosing, with vitamin A supplementation or even the ingestion of foods such as liver that are rich in vitamin A being contraindicated.

## Figures and Tables

**Table 1:** Prevalence ranges of vitamin A deficiency (VAD) in the population and their level of public health significance.

VAD as a Public Health Issue	
Public Health Significance (Degree of Severity)	Serum or Plasma Retinol <0.70 μmol/L in Preschool-Aged Children or Pregnant Women ^a^
Mild	≥2% to <10%
Moderate	≥10% to <20%
Severe	≥20%

**^a^** Source: Reference 46; Children 6–71 months of age. As there is no World Health Organization (WHO) recommended cut-off for serum retinol in pregnant women, the cut-off level for children was used (<0.70 μmol/L). The distribution of prevalence cut-offs for pregnant women is provisional.

**Table 2:** Suggested vitamin A supplementation scheme in pregnant women for the prevention of night blindness.

Target Group	Pregnant Women
Dose	Up to 10,000 IU vitamin A (daily dose) OR Up to 25,000 IU vitamin A (weekly dose)
Frequency	Daily or weekly
Route of administration	Oral liquid, oil-based preparation of retinyl palmitate or retinyl acetate
Duration	A minimum of 12 weeks during pregnancy until delivery
Settings	Populations where the prevalence of night blindness is 5% or higher in pregnant women or 5% or higher in children aged 24–59 months

IU: international units. Source: Reference 10.
